# Involvement of indoleamine 2, 3-dioxygenase (IDO) and brain-derived neurotrophic factor (BDNF) in the neuroprotective mechanisms of ferulic acid against depressive-like behaviour

**DOI:** 10.1007/s11011-023-01267-7

**Published:** 2023-07-25

**Authors:** Sanchari Basu Mallik, Jayesh Mudgal, Manas Kinra, Susan Hall, Gary D. Grant, Shailendra Anoopkumar-Dukie, Madhavan Nampoothiri, Yuqing Zhang, Devinder Arora

**Affiliations:** 1 School of Pharmacy and Medical Sciences, Griffith University, Gold Coast campus, Queensland, 4222 Australia; 2 Department of Pharmacology, Manipal College of Pharmaceutical Sciences, Manipal Academy of Higher Education, Manipal, 576104 India

**Keywords:** Ferulic acid, Depressive-like behaviour, Neuroinflammation, Cytokines, IDO, BDNF

## Abstract

Objective: Ferulic acid (FA) is a common food ingredient that is abundantly present in various routinely consumed food and beverages. Like many cinnamic acid derivatives, FA produces wide-ranging effects in a dose-dependent manner and various studies link FA consumption with reduced risk of depressive disorders. The aim of this study was to exploit the neuroprotective mechanisms of FA including indoleamine 2,3-dioxygenase (IDO), brain-derived neurotrophic factor (BDNF), and other pro-inflammatory cytokines by employing lipopolysaccharide (LPS)-induced depressive-like behaviour model. Methods: C57BL/6J male mice were divided into 4 groups consisting of saline (SAL), LPS, FA and Imipramine (IMI). Animals were pretreated orally with FA (10 mg/kg) and IMI (10 mg/kg) for 21 days once daily and all groups except SAL were challenged with LPS (0.83 mg/kg) intraperitoneally on day 21. Results: LPS administration produced a biphasic change in the behaviour of the animals where the animals lost a significant weight and express high immobility time at 24 h. Proinflammatory cytokines including, TNF-α, IL-6, IL-1β, and IFN-γ were significantly increased along with increased lipid peroxidation and reduced BDNF. Furthermore, the increased kynurenine to tryptophan ratio was indicative of elevated IDO activity. Conclusion: The results of this study emphasise that low dose of FA is effective in attenuating depressive-like behaviour by modulating IDO, BDNF and reducing neuroinflammation.

## Introduction

Ferulic acid (FA), 4-hydroxy-3-methoxycinnamic acid, is a natural phenolic compound that is extensively present in common fruits, vegetables, leaves and beverages like coffee and beer (Mancuso & Santangelo, 2014; Contardi et al. 2021; Stompor-Gorący & Machaczka, 2021). Being a polyphenol, the antioxidant properties of FA have been explored extensively (Graf 1992; Zduńska et al., 2018). Furthermore, it has been shown to be effective in depression (Singh et al. 2017; Liu et al. 2017; Zeni et al. 2017; Zheng et al. 2019), neuroinflammation (Sun et al. 2021; Jiang et al. 2021; Singh et al. 2017; Rehman et al. 2019), neurodegeneration (Chaudhary et al. 2019; Singh et al. 2017; Sultana 2012; Ojha et al., 2015), and cancer (Zduńska et al., 2018).

Inflammation plays an important role in the pathogenesis of major depressive disorders (MDD). The inflammation-induced depression theory is based on the studies where the patients undergoing cytokine therapy for infectious or autoimmune diseases expressed a relatively high incidence of depression, and physically healthy patients with depression showing elevated inflammatory markers (Zunszain et al. 2013; Jeon and Kim 2017). Moreover, clinical evidence suggests that inflammation alters the metabolism of essential amino acid tryptophan (Trp), which is the synthesis precursor of 5-hydroxytryptamine (5-HT) and plays an important role in the pathobiology of depression (Widner et al. 2002). The resulting increase in kynurenine (Kyn) to Trp ratio indicates the activation and involvement of indolamine 2,3-dioxygenase (IDO) enzyme (Widner et al. 2002). IDO is induced by proinflammatory cytokines, particularly by interferon-γ (IFN-γ) and tumor-necrosis factor-α (TNF-α) (Takikawa et al. 1999; Popov et al. 2006; Fujigaki et al. 2006). Administration of lipopolysaccharide (LPS), a bacterial endotoxin is a well-accepted tool to study neuroinflammation-induced sickness behaviour and depressive-like behaviour (Basu Mallik et al. 2016, 2021; Mudgal et al. 2019, 2020). Peripherally administered LPS induces the release of cytokines and causes long lasting inflammatory changes in both behavioural and brain biochemical markers (Basu Mallik et al. 2021; Mudgal et al. 2019).

Apart from cytokines, neurotrophins like brain derived neurotrophic factor (BDNF) and nerve growth factor (NGF), are associated with mood disorders (Castrén & Kojima, 2017; Wiener et al., 2015). Various human and animal studies link BDNF and neurological conditions including, Alzheimer’s disease (Nagata et al. 2014; Song et al. 2015), mood disorders (Nuernberg et al. 2016), schizophrenia (Islam et al. 2017), and Parkinson’s disease (Fischer et al., 2018). Moreover, proinflammatory cytokines downregulate the expression of neurotrophic factors in both cortex and hippocampus (Guan and Fang 2006). Similarly, there is a negative correlation between BDNF and proinflammatory cytokines including, IL-6) and interleukin-1β (IL-1β) (Jin et al. 2019; Lima et al. 2019).

Imipramine (IMI) is employed as a standard tricyclic (TCA) antidepressant molecule. Pharmacologically, IMI is well known to increase monoaminergic transmission by inhibiting the reuptake of both norepinephrine and serotonin through the reuptake proteins, the norepinephrine transporter (NET), and serotonin transporter (SERT). Various studies have supported the evidence that IMI inhibits proinflammatory cytokines production, microglial activation and stress-induced cortisol activation in depressed subjects (Antonioli et al. 2012; Ramirez and Sheridan 2016; Ramirez et al. 2015). Furthermore, it upregulates BDNF levels and inhibits IDO activation (Réus et al., 2013, Mohamed et al., 2013, Hall et al. 2016).

Based on the existing evidence, there are gaps in the literature about the in-vivo IDO modulatory role of FA. In this study, we have investigated the effects of FA on both peripheral and central IDO activity in LPS-induced model of depressive-like behaviour. Furthermore, this study also compiles the overall impact of FA on proinflammatory cytokines, oxidative stress, and neurogenesis in this model.

## Materials and methods

### Animals

Eight to ten weeks old C57BL/6J male mice weighing (20–30 g) were used in this study and were procured from Animal Resources Centre (Canning Vale, WA, Australia). All the animal experimental protocols were approved by the Institutional Animal Ethics Committee (IAEC) of Griffith University (PHM/01/15/AEC) and were performed in accordance with the guidelines set out in compliance with the National Institutes of Health Guide for Care and Use of Laboratory Animals (Publication No. 85 − 23, revised 1985). Animals were housed under controlled laboratory conditions, maintained at a 12 h day and night cycle with free access to food and water.

### Chemicals and reagents

Ferulic acid (FA), Imipramine (IMI), Lipopolysaccharide (LPS) (*Escherichia coli* serotype O111:B4), kynurenine, tryptophan, 2-thiobarbituric acid (TBA), protease inhibitor cocktail, sodium dihydrogen phosphate anhydrous, disodium hydrogen phosphate anhydrous, trichloroacetic acid and zinc acetate were purchased from Sigma-Aldrich (Sigma-Aldrich Co. LLC (St Louis, MO, USA). Acetonitrile (ACN, Scharlau, Spain), glacial acetic acid (GAC, Merck, Germany) and sterile 0.9% saline (Pfizer, West Ryde, Australia). All the other chemicals and reagents used in this study were of analytical grade.

### Drug treatments

**Fig. 1:**
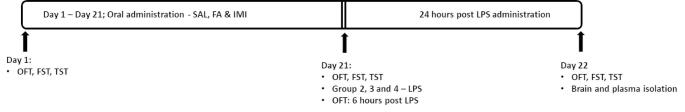
Schematic diagram of the drug treatment

Animals were divided into four groups (n = 6) and were randomised based on their body weight. Group 1 served as control (SAL); group 2 as LPS (SAL + LPS); group 3 ferulic acid (FA + LPS), and group 4 as imipramine (IMI + LPS). SAL and LPS groups were administered normal saline at a dose of 10 mL/kg, whereas FA and IMI groups were treated with FA (10 mg/kg) and IMI (10 mg/kg) respectively. All the drugs were administered by oral gavage (*p.o.*) using a dosing regimen of 21 days, whereas LPS was administered by intraperitoneal (*i.p.*) route on day 21. Animals were weighed daily for dosage calculation and the treatments were carried out between 08:00–09:00 once daily (Mudgal et al. 2019; Basu Mallik et al. 2021). One hour after the last dose of drug treatments, all the animals (except SAL group) received a single injection of LPS (0.83 mg/kg, i.p.). To ascertain the sickness-behaviour phase, open field test (OFT) was performed at 6 h post LPS administration. On day 22 OFT, forced swim test (FST) and tail suspension test (TST) were performed at 24 h post LPS administration for the observance of final depressive-like behaviour. Animals were euthanized as soon as the behavioural assays were completed and whole brain was isolated immediately and stored at -80 °C until further estimations (Fig. 1). Tissue samples were homogenised using chilled phosphate buffer (0.1 M, pH 7.4) for antioxidant and cytokine level estimations, and with trichloroacetic acid (1.6%) for the analysis of Trp and Kyn.

### Behavioural assays

The standard behavioural assays including, open field test (OFT), forced swimming test (FST) and tail suspension test (TST) were employed to assess the impact of LPS on locomotor activity (LMA) and behavioural despair. All the behavioural assays were performed by following the procedures detailed earlier (Basu Mallik et al. 2016; Mudgal et al. 2020). Briefly, the spontaneous LMA was assessed as number of square crossings in a plexiglass (40 × 40 × 40 cm) open field arena, which was divided in 10 × 10 cm virtual quadrants. FST was measured by the total immobility time over the last 5 min of the total 6 min of observational period in a transparent plexiglass cylinder (30 × 20 cm), whereas TST was assessed as the overall immobility time during the final 4 min of the total 5 min of observational period in a suspended phase from 15 cm height.

### Estimation of brain cytokine and lipid peroxidation levels

The levels of proinflammatory cytokines including, tumour necrosis factor-α (TNF-α), interleukin-6 (IL-6), interleukin-1β (IL-1β), interferon-γ (IFN-γ), malondialdehyde (MDA) (eBioscience, Cayman Chemical Company) and brain derived neurotrophic factor (BDNF) (Biosensis Pty Ltd.) were quantified using the commercially available enzyme-linked immunosorbent assay (ELISA) kits. All the biochemical estimations were performed following the manufacturer’s instructions. Total protein estimation was carried out using Pierce™ BCA Protein Assay Kit, as per manufacturer’s instructions.

### HPLC quantification of IDO activity

IDO activity was measured through the quantification of Trp and Kyn using high performance liquid chromatography (HPLC) as detailed earlier (Hall et al. 2016). The HPLC system consisted of a Shimadzu CBM-20 A Prominence communications bus control module, two Shimadzu LC-20AD UFLC liquid chromatograph pumps fitted with a solvent mixer, a Shimadzu DGU-20A3 Prominence degasser, a Shimadzu SIL-20 A HT UFLC Prominence chilled autosampler module, a Shimadzu CTO-20AC Prominence column oven, a Shimadzu SPD-M20A Prominence Diode array detector, and Lab solutions software. The column used for the analysis of Trp and Kyn was a Phenomenex Gemini (5 μm, 250 × 4.6 mm) reverse phase column (Phenomenex, Lane Cove, Australia) fitted with a Phenomenex Security Guard guard cartridge (Phenomenex).

Trp and Kyn were quantified using an isocratic method run over 10 min using 0.1% GAC (solvent A) and ACN (solvent B) (90:10% v/v) and 10 µL injections of sample were employed to quantify the analytes of interest. The UV absorbance was monitored at 360 nm (Kyn) and 275 nm (Trp).

### Statistical analysis

All the results were statistically analysed using GraphPad Prism 9 (Graph Pad Software Inc., San Diego, CA, USA). Results are represented as mean ± S.D. All the treatment groups were compared against the respective control groups (SAL or LPS) using one-way analysis of variance (ANOVA) followed by Dunnett’s multiple comparison test. “p” value of < 0.05 was considered to be statistically significant.

## Results

### Effects of FA and IMI on locomotion and immobility time

**Fig. 2:**
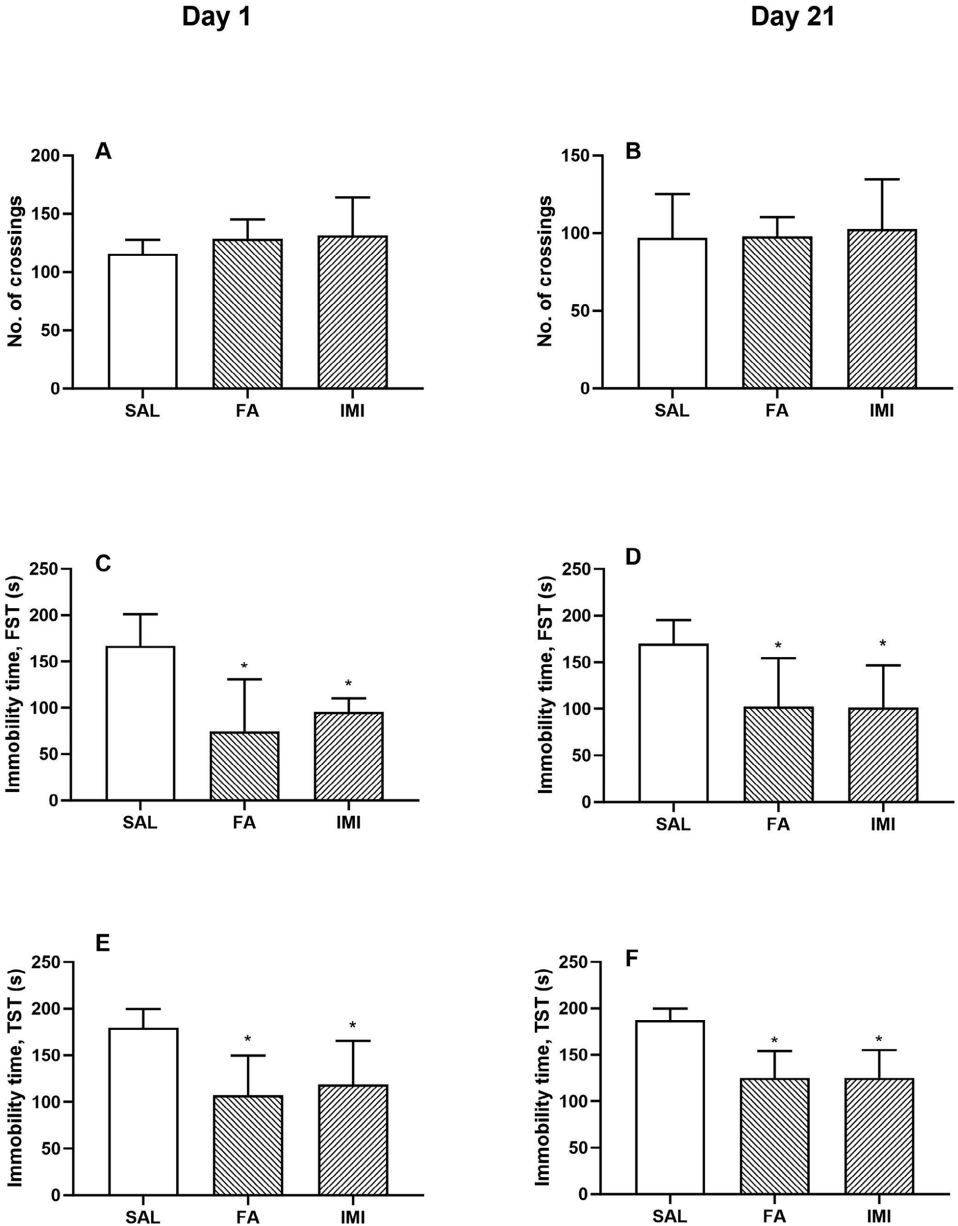
Effect of saline (SAL), ferulic acid (FA; 10 mg/kg) and imipramine (IMI; 10 mg/kg) on number of crossings **(A, B)**, immobility time (s), as observed in FST **(C, D)** and TST **(E, F)**. Values are the mean ± S.D. (n = 5), *p < 0.05 as compared to SAL group

The per-se effects of both FA and IMI were observed on day 1 and day 21. No significant effect on the ambulatory behaviour was observed in any treatment group on day 1 (115.60 ± 12.01 SAL, 128.20 ± 16.90 FA, 131.20 ± 32.69 IMI groups respectively, F [2, 12] = 0.69, p = 0.52; Fig. 2A), and day 21 (96.80 ± 28.41 SAL, 97.80 ± 12.40 FA, 102.60 ± 31.97 IMI groups respectively, F [2, 12] = 0.07, p = 0.93; Fig. 2B). Treatment of the animals with both FA and IMI significantly reduced the immobility time as observed by FST on day 1 (74.20 ± 56.59 FA, 95.60 ± 14.52 IMI vs. 167.00 ± 34.25 of SAL group respectively, F [2, 12] = 7.72; Fig. 2C), and day 21 (102.00 ± 52.33 FA, 101.40 ± 45.39 IMI vs. 169.80 ± 25.37 of SAL group respectively, F [2, 12] = 4.26; Fig. 2D). The immobility time observations from TST also showed a similar trend and a significant improvement was observed in both the treatment groups on day 1 (107.20 ± 42.40 FA, 118.80 ± 46.61 IMI vs. 179.60 ± 20.13 of SAL treatment groups respectively, F [2, 12] = 5.18; Fig. 2E), and day 21 (124.80 ± 29.09 FA, 125.40 ± 29.75 IMI vs. 187.40 ± 12.46 of SAL treatment groups respectively, F [2, 12] = 10.29; Fig. 2F).

### Effects of FA and IMI on depressive-like behaviour

**Fig. 3:**
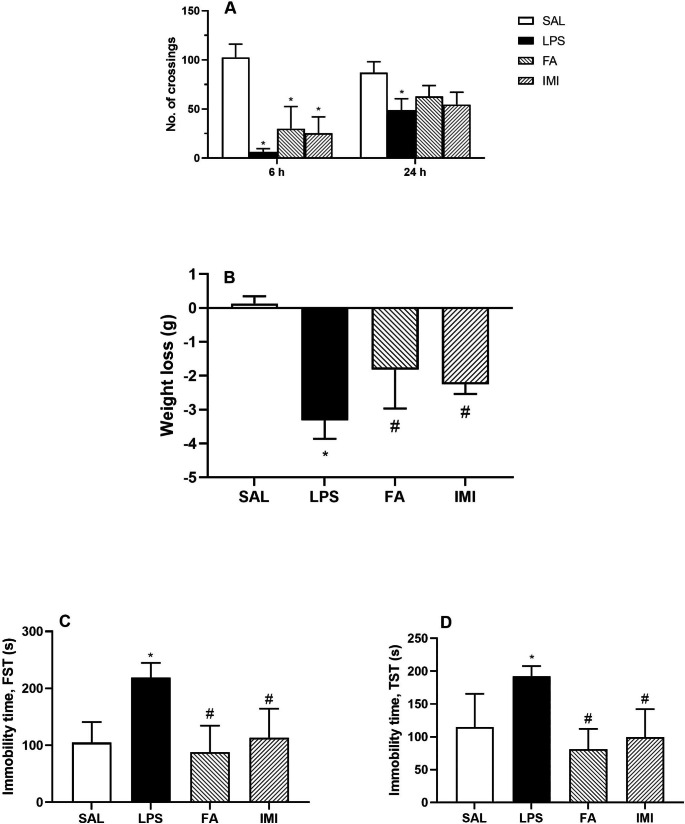
Effect of saline (SAL), ferulic acid (FA; 10 mg/kg) and imipramine (IMI; 10 mg/kg) on LPS-induced changes in locomotor activity at 6 and 24 h post LPS administration **(A)**, weight loss **(B)**, immobility time (s), as observed in FST **(C)** and TST **(D)** at 24 h post LPS administration. Values are the mean ± S.D. (n = 4–6), *p < 0.05 as compared to SAL group; #p < 0.05 as compared with LPS group

One hour after the last dose of SAL, FA and IMI, animals in group 2–4 were challenged with 0.83 mg/kg (*i.p.*) LPS on day 21. LPS administration significantly reduced the LMA at 6 h as observed by the total number of crossings in the open field arena (6.40 ± 3.29 vs. 102.60 ± 13.52 of SAL treated group). With passage of time the LMA had recovered significantly at 24 h post LPS administration (48.80 ± 11.71 vs. 6.40 ± 3.29 at 6 h post LPS, p < 0.05). Interestingly, neither of the treatment groups showed any significant improvement in the LMA at 6 h, or at 24 h post LPS administration (Fig. 3A).

Though the exploratory behaviour of the animals had improved within 24 h of LPS administration, however, it was accompanied with a significant loss of body weight (-3.32 ± 0.55 g vs. 0.13 ± 0.22 g of SAL treated group). Both FA and IMI pretreatments effectively prevented this LPS-induced loss of body weight (-1.82 ± 1.15 g FA, -2.25 ± 0.29 g IMI vs. -3.32 ± 0.55 g respectively, F [3, 20] = 28.48, Fig. 3B).

The decreased ambulation data was substantiated by the immobility times from both forced swim and tail suspension assays. Animals treated with LPS showed a significantly increased immobility time (219.00 ± 25.87 s vs. 104.80 ± 36.15 s of SAL treated group) in FST and (192.40 ± 15.36 s vs. 114.80 ± 50.63 s of SAL treated group) in TST. Chronic treatment with FA significantly improved the immobility time in both FST (88.20 ± 46.30 s, F [3, 17] = 10.79, Fig. 3C), and TST (81.20 ± 31.01 s, F [3, 16] = 8.57, Fig. 3D). A similar significant improvement was also observed with IMI treatment (113.60 ± 50.76 s FST and 99.60 ± 42.65 s TST respectively).

### Effect of FA and IMI on brain inflammatory markers and MDA levels

**Fig. 4:**
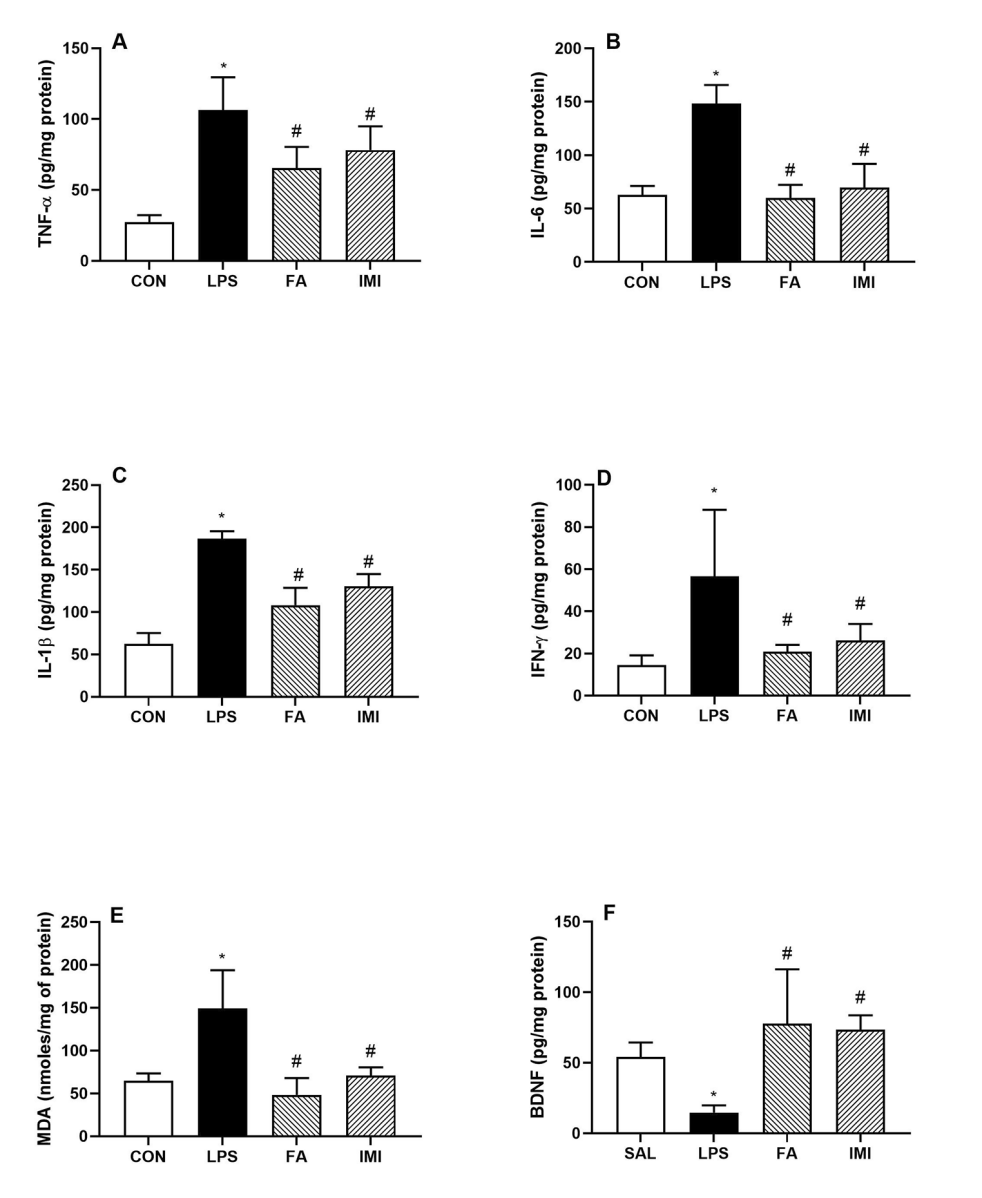
Effect of saline (SAL), ferulic acid (FA; 10 mg/kg) and imipramine (IMI; 10 mg/kg) on LPS-induced changes in brain homogenates: TNF-α (pg/mg protein) **(A)**, IL-6 (pg/mg protein) **(B)**, IL-1β (pg/mg protein) **(C)**, IFN-γ (pg/mg protein) **(D)**, MDA levels (nmoles/mg protein) **(E)** and BDNF (pg/mg protein) **(F)**. Values are the mean ± S.D. (n = 4–6), *p < 0.05 as compared to SAL group; #p < 0.05 as compared with LPS group

The major proinflammatory cytokines including, TNF-α, IL-6, IL-1β and IFN-γ were quantified in the brain homogenates (pg/mg protein). TNF-α (106.50 ± 23.12); IL-6 (148.40 ± 17.30); IL-1β (186.60 ± 8.94) and IFN-γ (56.61 ± 31.62) were found to be significantly elevated at 24 h post LPS administration as compared to SAL treated animals. Both FA and IMI pretreatments were able to counteract the impact of LPS-induced changes in these cytokines; TNF-α (65.43 ± 15.04 FA and 78.19 ± 16.79 IMI, F [3, 16] = 20.23, Fig. 4A), IL-6 (59.87 ± 12.45 FA and 69.52 ± 22.40 IMI, F [3, 16] = 34.99, Fig. 4B), IL-1β (108.20 ± 20.51 FA and 130.50 ± 14.32 IMI, F [3, 15] = 51.40, Fig. 4C), IFN-γ (20.90 ± 3.25 FA and 26.26 ± 7.74 IMI, F [ 3, 15] = 6.27, Fig. 4D).

Similarly, lipid peroxidation (as measured by MDA levels) was significantly increased by LPS (149.40 ± 44.52 vs. 64.71 ± 8.69 of SAL treated group). And, both FA and IMI reduced this effect of LPS at the dose and regimen used in this study (48.38 ± 19.62 FA and 70.70 ± 9.86 IMI, F [3, 16] = 15.98, Fig. 4E).

### Effect of FA and IMI on brain BDNF

BDNF levels (pg/mg protein) were significantly reduced by LPS administration (14.74 ± 5.08 vs. 54.16 ± 10.34 of SAL treated group). Pretreatment with both FA and IMI protected this decline in BDNF levels in both the groups (77.87 ± 38.39 FA and 73.44 ± 10.21, F [3, 15] = 9.22, Fig. 4F).

### Effect of FA and IMI on brain kyn and trp levels

**Fig. 5:**
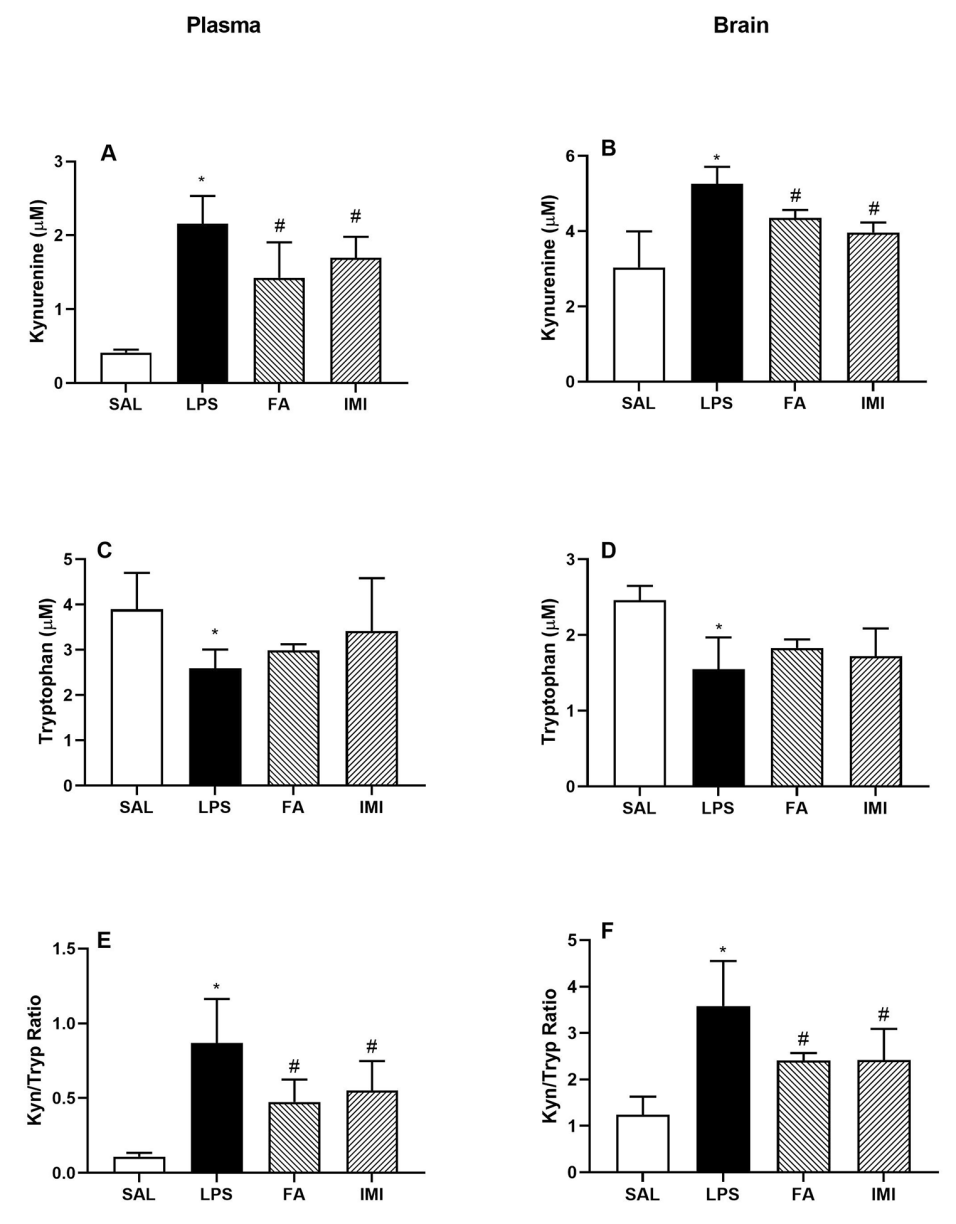
Effect of saline (SAL), ferulic acid (FA;10 mg/kg) and imipramine (IMI; 10 mg/kg) on LPS-induced changes in the plasma **(A**, **C** and **E)** and brain homogenates **(B**, **D** and **F)**: Kyn (µM) **(A** & **B)**, Trp (µM) **(C** & **D)**, Kyn/Trp ratio **(E** & **F)**. Values are the mean ± S.D. (n = 4–5), *p < 0.05 as compared to SAL group; #p < 0.05 as compared with LPS group

LPS administration significantly increased the Kyn levels (µM) in the plasma (2.16 ± 0.38 vs. 0.41 ± 0.04 of SAL treated group) and brain homogenates (5.26 ± 0.46 vs. 3.04 ± 0.96 of SAL treated group). Both FA and IMI pretreatment groups expressed a significant reduction in these levels (1.43 ± 0.48 FA and 1.70 ± 0.28 IMI, F [3, 16] = 24.03, in the plasma and 4.35 ± 0.22 FA and 3.97 ± 0.26 IMI, F [3, 15] = 13.10 in the brain, Fig. 5A and B respectively). Trp levels (µM) were significantly reduced in LPS treated group in the plasma (2.60 ± 0.41 vs. 3.90 ± 0.80 of SAL treated group) and in brain homogenates (1.55 ± 0.42 vs. 2.46 ± 0.19 of SAL treated group). Neither of the treatment groups showed any improvement in the Trp levels (Fig. 5C and D respectively). Interestingly, the Kyn to Trp ratio was significantly reduced by both FA and IMI in both plasma (0.47 ± 0.15 FA and 0.55 ± 0.20 IMI vs. 0.87 ± 0.29 of LPS treated group, F [3, 16] = 13.05, Fig. 5E) and brain (2.41 ± 0.16 FA and 2.42 ± 0.67 IMI vs. 3.58 ± 0.97 of LPS treated group, F [3, 14] = 9.88, Fig. 5F).

## Discussion

LPS upregulates the transcription factors like nuclear factor- κB (NF-κB) and interferon-regulatory factors (IRFs) by activating Toll Like Receptor 4 (TLR4) and thereby induces various proinflammatory cytokines and IFNs (Laird et al. 2009; Williams et al. 2006; Dorrington and Fraser 2019; Kinra et al. 2021). When an inflammatory milieu is created within the systemic circulation by the administration of bacterial endotoxin LPS, it produces an array of behavioural and biochemical changes in a time-dependent manner. The initial phase of first 2–4 h is the “sickness behaviour”, where the animals mainly express severe lack of exploratory behaviour, bradykinesia, increased body temperature and lack of appetite (Painsipp et al. 2011; O’Connor et al., 2009). These behavioural changes directly correlate with the acute upregulation of plasma proinflammatory cytokine levels, and as their levels start to decline, the symptoms of sickness behaviour gradually wane off. However, the feeding and exploratory behaviour improves within 24 h post-LPS administration and a second phase ensues, that expresses the long-term “depressive-like behaviour” in mice with the immobility status still predominantly expressed (Painsipp et al. 2011; O’Connor et al., 2009; Basu Mallik et al. 2021). It is presumed that this depressive-like behaviour is linked with the long-lasting changes in the neuroinflammatory response, since it appears roughly at the time point when the plasma levels of proinflammatory cytokines have fallen to the basal level (Andreasen et al. 2008; Lotter et al. 2006; Qin et al., 2007). In order to confirm that all the animals expressing the depressive-like behaviour have passed through the initial sickness-behaviour, the spontaneous LMA was observed at both 6 and 24 h post LPS treatment. The exploratory behaviour was significantly reduced at 6 h. On the other hand, at 24 h, the animals expressed an improved LMA, a significant loss of weight, and accompanied with increased immobility time in both FST and TST. Chronic pretreatment of the animals with both FA and IMI was unable to improve the spontaneous locomotion in both sickness-behaviour and depressive-like behaviour at both 6 and 24 h respectively. The overall LMA data indicates that FA does not produce any stimulatory effect and the immobility data from FST and TST cannot be linked with this activity. On the other hand, in depressive-like state both FA and IMI groups lost significantly less weight and expressed significantly improved immobility time in the FST and TST.

In our earlier studies, we have reported that the plasma levels of TNF-α and IL-6 increase rapidly with a single *i.p.* injection of LPS, and these cytokines can be quantified in the brain tissue (Basu Mallik et al. 2016; Mudgal et al. 2019, 2020). Plasma TNF-α levels have been shown to peak within 1.5-2 h, whereas IL-6 and IL-1β peak in 4–6 h post LPS administration in both mice and humans (Andreasen et al. 2008; Lotter et al. 2006; Liu et al. 2017). A single administration of 0.83 mg/kg, i.p. of LPS produced a significant increase in the major proinflammatory cytokines including, TNF-α, IL-6, IL-1β, and IFN-γ in the brain within 24 h. Under normal circumstances, the tight junctions of blood-brain-barrier (BBB) consisting of astrocytes and capillary endothelial cells guards the brain from systemic pathogens (Zhao et al. 2015; Abbott et al. 2010). However, this brain protection is compromised with severe systemic inflammation and moreover, LPS directly leads to release of proinflammatory mediators in the CNS (Jeltsch-David and Muller 2016). Furthermore, there is direct activation of cytokine receptors including, IL-1β, IL-6 and TNF-α, on the cerebral endothelium (Skelly et al. 2013; Varatharaj and Galea 2017). In all the LPS-treated animals, pretreatment with FA and IMI significantly reduced the inflammatory cytokines along with the concomitant oxidative stress. In our present study we explored the involvement of IDO enzyme in this model of depressive-like behaviour. Koshiguchi et al. (2017) has shown that FA suppresses the mRNA expression of IDO in microglial cells, however, there is lack of evidence for such effects of FA in the in-vivo conditions (Koshiguchi et al. 2017; Badawy and Guillemin 2019). The increased mRNA expression is not a true indication of increased enzyme activity, and plasma Kyn/Trp ratio is taken as a reflection of extrahepatic IDO activity (Badawy and Guillemin 2019). Under basal metabolic conditions, IDO does not play an active role in Trp metabolism and the Kyn/Trp ratio is very low and is almost undetectable, however, immune activation during inflammatory conditions upregulates IDO and significantly enhances this ratio. The principal effector of IDO is IFN-γ (Pfefferkorn et al. 1986; Werner et al. 1987). The proinflammatory cytokines IL-1β and TNF-α potentiate IDO induction by IFN-γ, even though they do not have a major effect of their own (Badawy 2013). Activation of this enzyme deviates the metabolism of Trp more towards Kyn pathway and less on the serotonin and melatonin side. As seen in the results, and consistent with literature, LPS treatment significantly depleted the plasma and brain Trp levels and elevated the Kyn levels, with an overall increase in Kyn/Trp ratio. Pretreatment with FA and IMI attenuated these LPS-induced changes and significantly reduced the Kyn levels. It is worth noticing that the levels of Trp were not restored in any of the pretreatment groups, suggesting that FA and IMI mainly reduce the IDO activation and impact the Kyn/Trp ratio significantly.

LPS administration also led to a significant depletion in the protective growth factor, BDNF. BDNF has been shown to be beneficial for neuronal function under stress conditions (Benarroch 2015). Furthermore, several studies indicate towards a reduced BDNF signaling in depression and improvement with the antidepressant drug treatment (Lee and Kim 2010; Jin et al. 2019). Pretreatments with both FA and IMI significantly increased BDNF levels in LPS administered animals. In retinal cell suspensions, FA has shown better results on nerve cell proliferation as compared to BDNF (Li et al. 2003). Moreover, oral administration of FA promoted neurogenesis and increased cAMP response element binding protein (CREB) phosphorylation and BDNF mRNA levels in cortisone-induced depressive-like behaviour in mice (Yabe et al. 2010). Similarly, IMI induces BDNF expression in cultured astrocytes and after chronic treatment in rodents and clinical subjects (Réus et al., 2013, Antonioli et al. 2012).

In conclusion, it is noteworthy that chronic oral administration of FA protected against the LPS-induced neuroinflammation. Various studies have proposed a similar protective mechanism of FA in different stress-induced depressive-like behaviour studies, however, this study compiled various plausible mechanisms by which FA is involved in providing a neuroprotective effect in depressive-like behaviour in mice. Though, the exact status of FA and other relevant potential candidates of natural origin in prevention or treatment of human depression is not clear, however, this experimental approach supports the antidepressant properties of FA, when consumed in moderate doses. Further clinically oriented chronic studies may enlighten the neuro-modulatory potential and therapeutical role of FA.

## Data Availability

The datasets generated during and/or analysed during the current study are available from the corresponding author on reasonable request.
